# 

**Published:** 1894-07-21

**Authors:** 


**Notices of Births, Deaths, and Marriages** are inserted in this column at a charge of 2s. 6d. for an announcement not exceeding four lines.

**Notice to Advertisers and Subscribers.**

All Advertisements, Orders for Copies of The Hospital, and Business Communications should be addressed to **The Publisher, NOT TO THE EDITOR**, The Hospital, 428, Strand, W.C.

The Cost of Subscription, post free, is as follows:—Per week, 2½d.; per quarter, 2s. 8d.; per half-year, 5s. 3d.; per annum, 10s. 6d. Foreign:—Per Annum, 12s. 8d.; payable, in all cases, in advance.

### NOTICE TO CORRESPONDENTS.

All MS., Letters, Books for Review, and other matters intended for the Editor should be addressed The Editor, The Lodge, Porchester Square, London, W.

The Editor cannot undertake to return rejected MS., even when accompanied by stamped directed envelope.

**"Burdett's Hospital Annual," 1894.**

Fifth year of publication. 900 pp. Boards, gilt lettering. A most complete guide to British, Colonial, Indian, and American Hospitals, Dispensaries, Nursing and Convalescent Institutions, and Asylums. Price 5s. Published by The Scientific Press (Limited), 428, Strand, W.C.

**NOTICE.—The Index for Vol. XV. (ending March 31st, 1894) is on sale at the Office of this Journal, price 2½d., post free.**

### SCRAPS AND GLEANINGS.

The Evesham Joint Hospital Board have decided to abolish all compulsory fees for the maintenance of patients in the Sanatorium.

The amount collected this year in the London synagogues by the Chief Rabbi in aid of the Metropolitan Hospital Sunday Fund was £832 1s. 3d.

Messrs. Rothschild have sent a donation of 200 guineas to the British Home for Incurables, to assist in opening the new home free of debt.

The Belgrave Hospital for Children, Gloucester Street, Pimlico, have received a grant of thirty guineas from the Corporation of the City of London.

The foundation-stone of the Convalescent Home in connection with the Charing Cross Hospital was laid last week at Limpsfield Chart, Surrey.

The Cornelia Hospital at Poole has been re-opened, having been closed for some weeks during repair. The whole arrangement of the hospital is immensely improved.

A charmingly situated cottage hospital has been erected on the high ground of East Hill, near Dartford. The wards will be ready for occupation in a few weeks' time.

The directors of the proposed new infirmary at Paisley are making an urgent appeal for funds. The sum of £15,000 is still required to enable the building to be proceeded with.

The opening ceremony in connection with the new dispensary of the Glasgow Eye Infirmary was performed in presence of a large company. The new buildings are built in Renaissance style.

The *Edinburgh Scotsman* reports a meeting held in protest against the erection of a small-pox hospital in the Cowgate, at Leith. It was decided that a deputation should present a protest to the Council.

The Middlesex Hospital has received a donation of £1,000 from Mr. John Maple, through the medium of Sir Blundell Maple. The gift was presented as a token of goodwill towards the institution.

The Prince and Princess of Wales visited Rhyl on Friday, 13th of this month, in order that the Princess might lay the foundation-stone of a Convalescent Home for Children, which is to be put up at a cost of £15,000.

At the meeting of the Council of the Royal College of Surgeons last week, Mr. J. Whittaker Hulke, F.R.S., was re-elected President of the College, and Mr. R. Harrison and Mr. A. Willett were appointed Vice-Presidents.

The annual Congress of the Institute of Public Health will be opened at the Guildhall on July 26th, closing on July 31st. A circular has been sent out to local authorities inviting delegates, and a large meeting is anticipated.

The plans for a new hospital at Birmingham for the reception of scarlet fever cases have been laid before the principal medical officer of the Local Government Board. The cost of the new building is estimated at £30,000.

The new paddle steamer built to the order of the Metropolitan Asylums Board for carrying patients to the hospital ships in Long Reach has made her official trial trip on the Thames. The trial proved most satisfactory in all respects.

The procession and fête held at Swindon on Hospital Saturday in aid of the Swindon Victoria Hospital was an undoubted success. An extensive programme of amusements was provided, and the gate-money alone amounted to £33 11s.

At the annual meeting of the Ipswich and East Suffolk Hospital the financial statement showed a deficit of £499 17s. 9½d. It was agreed to re-elect the Committee of last year to decide what course to adopt to obtain further subscriptions.

Mr. Ernest Hart, Chairman to the National Health Society, has been elected honorary president of the third section of the eighth International Congress of Hygiene and Demography. The congress is to be held at Budapest in September.

The suggestion has been made to turn the interesting ruin of "Castle Ravensheuch," near Kirkcaldy, into a hospital for infectious diseases. The position of the castle is an ideal one for the purpose in view, being isolated, perfectly dry, and well placed for drainage.

The first demonstration ever held in Martham in aid of the Hospital Sunday Fund, took place this year under the banner of the local Friendly Societies. Services were held in St Mary's Church and the Primitive Methodist Chapel, the two collections amounting to £7 7s.

The new union infirmary at Dewsbury has been opened. The pile of buildings have cost about £21,000. The Dewsbury Board of Guardians have provided four homes for the children obliged to come to the workhouse. These children's homes have been provided at the cost of £2,500.

At the annual meeting at Louth of the governors and subscribers to the Louth Hospital and Dispensary, the financial statement showed a considerable balance on the wrong side. It was stated that the funds received from the collections on Hospital Sunday amounted to much less than those of last year.

The annual public collections in aid of the North Staffordshire Infirmary and the Hayward Hospital were taken in all the towns of the Potteries on the last Saturday in June. The results were most satisfactory. There was a sale of flowers held by ladies in St. John's Square and Swan Square.

At the annual meeting of the Yarmouth Hospital the committee stated that there was a great want of accommodation in the hospital for female in-patients. They would therefore appeal to the public to furnish them with funds sufficient to enable them to convert the present out-patients' department into a ward for women, and build convenient premises for out-patients.

The thirteenth annual report of the Sarah Nicol Memorial Cottage Hospital, at Llandudno, has just been published. The financial position shows a balance of £45 14s. 5d. The Cottage Hospital has no secured income, and the Board of Management urgently call upon their supporters to aid them in opening an endowment fund, the building having been supported for the last thirteen years by voluntary contributions, a position which necessarily entails a great deal of anxiety to the managers.

### THE STUDENT OF MEDICINE.

### APPOINTMENTS.

E. Atkinson, M.R.C.S.Eng., appointed Consulting Surgeon to the Leeds General Infirmary. R. de la Poer Beresford, M.D.Glas., L.R C.P., L.R.C.S.Edin., reappointed Medical Officer of Health to the Oswestry Town Council. C. Y. Biss, M.A., M.D.Cantab., F.R.C.P., appointed Physician to the Hospital for Consumption and Diseases of the Chest, Brompton. H. M. Dowler, M.R.C.S., L.R.C.P.Lond., appointed Junior House Surgeon to the Westminster Hospital. G. M. Edwards, B.A., M.B., B.C.Cantab., M.R.C.S.Eng., L.R.C.P.Lond., appointed House Surgeon to the Middlesex Hospital. Foster, M.B., L.R.C.P.Lond., M.R.C.S.Eng., appointed House Physician to the Leicester Infirmary. J. Fuller, F.R.C.S.Edin., M.R.C.P.I., appointed Medical Officer of the Workhouse of the Bedminster Union. R. N. Hartley, M.B., B.S., M.R.C.S., appointed Honorary Surgeon to the Leeds General Infirmary. J. S. Hill, M.B., C.M., appointed Honorary Surgeon to the Royal South London Dispensary. Mr. A. Lavery, appointed Assistant House Surgeon to the Coventry and Warwickshire Hospital, Coventry. G. H. Nowell, B.A., B.C., M.B.Cantab., L.S.A.Lond., appointed Senior House Surgeon to the Westminster Hospital. J. S. Owens, B.A., M.B., B.Ch., appointed Medical Officer of the Gorey Dispensary District. C. E. Purslow, M.D.Lond., M.R.C.P.Lond., appointed Ingleby Examiner, Queen's Faculty of Medicine, Mason College, Birmingham, for the current year. H. J. Robinson, M.R.C.S., L.R.C.P.Lond., appointed Medical Officer and Public Vaccinator for the Burnley District and the Workhouse of the Burnley Union. S. Saunders, M.D., L.R.C.P., M.R.C.S., reappointed Medical Officer of Health for the City of London. S. K. Smith, F.R.C.S., appointed Surgeon to the Liverpool Stanley Hospital. R. S. Thomas, M.A., M.B., B.C.Cantab., M.R.C.S.Eng., L.R.C.P.Lond., reappointed Casualty Surgical Officer to the Middlesex Hospital. W. A. Ward, M.R.C.S.Eng., L.R.C.P. Lond., L.S.A., reappointed Casualty Medical Officer to the Middlesex Hospital.

### VACANCIES.

The following vacancies are announced this week, the amount of salary in each case being given after the name of the institution:

### Resident Medical Officers.

Cambridge Lunatic Asylum, Fulbourn, near Cambridge.—Assistant Medical Officer. Salary £140 per annum, with board, lodgings, and attendance in the Asylum. Applications to T. Musgrave Francis, Clerk to the Visitors, by July 21st.

Chichester Infirmary.—House Surgeon. Salary £80 per annum, with board, lodging, and washing. Applications to Eugene Street, Secretary, by August 6th.

East London Hospital for Children and Dispensary for Women, Glamis Road, Shadwell, E.—Resident Medical Officer. Must be registered practitioner in medicine and surgery. Salary £80 per annum, with board and residence. Applications and testimonials to Thomas Hayes, Secretary, by July 23rd.

Manchester Royal Infirmary. — Resident Surgical Officer; doubly qualified, unmarried, and not less than 25 years of age. Salary £150 per annum, with board and residence. Applications to W. L. Saunder, General Superintendent and Secretary, by July 28th.

North Staffordshire Infirmary and Eye Hospital, Hartshill, Stoke-upon-Trent.—House Physician. Salary £100 per annum, increasing £10 per annum at the discretion of the committee, with furnished apartments, board, and washing. Applications to the Secretary by July 23rd.

Seamen's Hospital Society, "Dreadnought."—Junior House Surgeon, doubly qualified, for Branch Hospital, Royal Victoria and Albert Docks, E. Salary £50 per annum, with board and residence. Applications to P. Michelli, Secretary, Seamen's Hospital Society, Greenwich, S.E., by July 30th.

### THE STUDENT OF MEDICINE—*continued.*

Sussex County Hospital, Brighton.—House Surgeon; doubly qualified, unmarried, and under 30 years of age. Salary £120, rising to £140 per annum, with board, residence, and washing. Applications to the Secretary by August 7th.

### Non-Resident Medical Officers.

Charing Cross Hospital, Strand, W.C. — Assistant Surgeon; must be F.R.C.S.Eng., and reside within three miles of the hospital. Applications to the Chairman of the Committee of Selection by July 28th.

West London Hospital, Hammersmith Road, W. — Dermatologist. Applications to R. J. Gilbert, Secretary and Superintendent, by August 1st.

### PASS LISTS OF THE UNIVERSITIES AND COLLEGES.

### University of Edinburgh.

The following gentlemen have passed the final examination for the degrees of M.B. and C.M. (Those marked with an asterisk have passed "with distinction.") W. D. Adams, M.A.; C. H. Adamson, C. C. Aitken, C. J. H. Aitken, G. N. Alexis, *S. H. B. Allison, B.A.; J. W. Anderson, R. Y. Anderson, J. C. Atkinson, C. Ayres, B.A.; A. Balfour, T. M. Bartlett, H. Bateson, *J. M. Beattie, B.A.; *R. W. Beesley, W. Bethune, R. St. G. S. Bond, W. H. Bowie, A. Bremner, R. W. Briggs, B. W. Broad, H. R. Brown, M.A.; R. T. Bruce, W. H. Bryce, M. Burnet, D. J. S. Burt, A. J. Campbell, *A. K. Campbell, C. S. Camtrell, O. H. Chapman, P. F. Chapman, R. M. Clark, R. Cochrane, M.A.; J. A. Coutts, W. B. Craig, M.A.; D. Macforn, R. Crichton, H. M. Crosby, A. Dall, M.A.; R. T. Davidson, T. H. C. Durham, G. A. Dickson, E. R. Dodds, E. A. W. English, T. Evans, J. H. Ewart, F. H. Fairweather, R. W. Fell, J. Fenton, P. J. H. Ferguson, J. S. Flett, M.A., B.Sc.; J. V. Forrest, *E. J. H. Fraser, S. Fraser, W. C. Fraser, *G. B. French, J. W. Geddes, A. J. Gibson, J. A. Gibson, *T. Gibson, M.A.; H. H. Gill, *J. R. Gilmour, J. O. Goldie, A. D. M. Grant, *L. Grant, W. Y. Grant, G. D. Gray, J. Gray, H. M. Green, J. D. Gregodson, R. Grieve, W. Haig, F. H. Hardman, J. Henderson, R. Z. Herdmau, J. E. Hewlitt, A. Heys, E. R. Holmes, G. Hudson, P. T. Hughes, J. K. Jamieson, T. H. Jamieson, D. A. Johnstone, W. E. Johnstone, M.A.; J. H. Jones, T. H. Jones, J. W. Keighly, B. C. Kelly, T. B. Kenny, A. E. Kidd, D. F. Laidlaw, G. C. Lang, J. A. Laing, J. Lawrie, R. E. Legat, G. F. Leicester, W. Leslie, J. M. Lincoln, *T. G. Lusk, D. G. MacArthur, A. J. Macdougall, C. J. R. MacFadden, J. A. Macfarlane, A. J. M'Ilroy, J. Maciver, M. M'Larty, J. F. Macphail, M.A.; J. F. Macpherson, *J. D. Macrae, C. M'Vicar, M.A.; W. Mailor, H. B. Mapleton, B.A.; H. H. Marshall, W. R. Martine, F. O. N. Mill, J. M. Menzies, S. Messulam, A. Mitchell, W. Mitchell, K. W. Monserrat, E. C. Moore, R. O. Morris, M.A.; R. S. Mowat, J. R. Muir, H. V. Munster, G. A. Murray, T. M. Mair, J. Nicolson, F. R. Oliphant, D. Orr, C. W. Owen, A. A. Palmer, R. Parkhurst, J. A. Parsons, G. W. S. Paterson, T. B. Pearson, T. C. Penfold, A. P. Percival, J. M. Pereird, T. Pettey, C. E. Potter, D. R. Price, A. M. M. Pringle, F. G. Proudfoot, M.A.; E. de C. Prout, P. M. Ragg, A. Reid, W. Riach, J. K. Richards, A. Richardson, W. H. Robertson, A. Rodger, L. Rose, M.A.; H. Ross, G. H. R. Harrison, J. M. Rutherford, J. Scott, T. M. Scott, P. J. Sharp, G. Shaw, T. D. S. Shaw, T. R. S. Sibbald, C. M. Simpson, S. S. Skinner, B.A.; P. D. Smith, R. W. I. Smith, T. Smith, W. E. Smith, A. M. Stafford, J. G. Standing, *W. A. Stephen, M.A.; M. B. Steuart, H. Steven, W. L. Stevenson, *J. P. Stewart, M.A.; *W. S. Strapp, J. Struthers, F. W. Taylor, W. S. S. Titterton, M. L. M. Vaudin, G. D. Waal, J. S. H. Walker, J. W. T. Walker, F. T. Walmsley, F. Ward, J. Watson, E. C. Watts, E. T. Whitaker, A. Whytt, E. D. Williams, J. M. Wishart, J. J. N. Wood, H. M. Woodhead, R. J. T. Wright, M.A.; and G. P. Yule.

SEAMEN'S HOSPITAL SOCIETY,
"DREADNOUGHT."

SUSSEX COUNTY HOSPITAL.

